# *Heterorhabditis* and *Photorhabdus* Symbiosis: A Natural Mine of Bioactive Compounds

**DOI:** 10.3389/fmicb.2022.790339

**Published:** 2022-03-29

**Authors:** Ripu Daman Parihar, Urvashi Dhiman, Anil Bhushan, Prashant Kumar Gupta, Prasoon Gupta

**Affiliations:** ^1^Department of Zoology, University of Jammu, Jammu, India; ^2^Department of Zoology, DAV University, Jalandhar, India; ^3^Natural Products and Medicinal Chemistry Division, Council of Scientific and Industrial Research (CSIR)-Indian Institute of Integrative Medicine, Jammu, India; ^4^Academy of Scientific and Innovative Research (AcSIR), Ghaziabad, India; ^5^Department of Horticulture, Rajmata Vijayaraje Scindia Krishi Vishwa Vidyalaya, Gwalior, India

**Keywords:** *Heterorhabditis*, *Photorhabdus*, nematodes, antimicrobial, antiprotozoal, anti-inflammatory and anticancer

## Abstract

Phylum Nematoda is of great economic importance. It has been a focused area for various research activities in distinct domains across the globe. Among nematodes, there is a group called entomopathogenic nematodes, which has two families that live in symbiotic association with bacteria of genus *Xenorhabdus* and *Photorhabdus*, respectively. With the passing years, researchers have isolated a wide array of bioactive compounds from these symbiotically associated nematodes. In this article, we are encapsulating bioactive compounds isolated from members of the family Heterorhabditidae inhabiting *Photorhabdus* in its gut. Isolated bioactive compounds have shown a wide range of biological activity against deadly pathogens to both plants as well as animals. Some compounds exhibit lethal effects against fungi, bacteria, protozoan, insects, cancerous cell lines, neuroinflammation, etc., with great potency. The main aim of this article is to collect and analyze the importance of nematode and its associated bacteria, isolated secondary metabolites, and their biomedical potential, which can serve as potential leads for further drug discovery.

## Introduction

Bioactive compounds represent substances having biological activity, which mediates some metabolic process leading to better health (Solomon and William, 2003; Bender, 2009). Nature is a reservoir of new bioactive compounds also known as natural products (NPs), whose study is indispensable for drug discovery and development (Ebada et al., 2008; Shen, 2015). These NPs are produced by plants, microorganisms, and animals (Baker et al., 2000) and symbiotic microorganisms. In developing countries, these NPs play an intrinsic role in the life of human beings substituting medicines due to easy availability and low cost. These NPs display a wide range of structural diversity, which correlates with biological activity like antitumor agents and enzyme inhibitors, antibiotics, immunosuppressive agents, growth promoters, herbicides, insecticides, and antiparasitic agents (Carmichael, 1992; Méndez and Salas, 2001). More than 300,000 NPs exist in literature and are mostly classified into five broader categories including alkaloids, steroids, terpenoids, polyketides, and fatty acid-derived substances, shikimate-derived compounds, and non-ribosomal polypeptides (Méndez and Salas, 2001). Among 10,000 biologically active compounds, almost 8,000 are antitumor and antibiotic agents (Brusotti et al., 2014). Since 1928, the year of penicillin discovery, around 20 different classes of antibiotics have been routed to the market (Coates et al., 2002; Powers, 2004). Most of the classes were explored from 1940 to 1962 and protected us from various infections for around 50 years (Coates et al., 2011). Recently, it has been reported that there are around 450,000 NPs out of which 70% are from plant origin (Ntie-Kang and Svozil, 2020). Over the years, the race to explore new biotics for controlling diseases is countered by pathogen resistance following Darwin’s principle. Utility of bioactive compounds in diverse commercial sectors like food, pharmaceutical, and chemical industries indicates the need to explore novel sources of bioactives.

Different living organisms produce a wide array of natural products. They are reported from both eukaryotic and prokaryotic (Prokaryotae, Monera) organisms but the ability to produce secondary metabolites is not uniform in all the species. Unicellular bacteria, filamentous actinomyces, and fungi frequently produce a wide array of secondary metabolites. Over few decades, the races to isolate useful molecules have shown established animal kingdom as a new and rich source of bioactive metabolites. These have been reported from a few marine invertebrates from Porifera, Cnidaria, Anthozoa, Tunicates, Mollusca, Echinodermata, etc. (Cabeza et al., 2021; Chamika et al., 2021; Mayefis and Widiastuti, 2021; Ramesh et al., 2021; Riccio et al., 2021; Sibiya et al., 2021; Singh et al., 2021; Tsvetkov et al., 2021; Wewengkang et al., 2021). The number of bioactive compounds from marine sources has been increasing linearly, i.e., 25 in 1972, 300 in 1982, 1,500 in 1992, and over 6,000 today, and more than 3,300 compounds are reported from sponges only (Bérdy, 2005). Besides this, certain animal microbes (like protozoa and ciliates), worms, insects, amphibians, and some higher vertebrates have been reported to produce bioactive compounds raising their number above 43,000. It includes antimicrobial, antitumor, products from marine sources, and microbial metabolites (Bérdy, 2005; Abd-Elgawad, 2021). In this review, we are presenting the genus *Heterorhabditis*, a less explored source of bioactive compounds from the gut of entomopathogenic nematode (EPNs), i.e., Heterorhabditidae. This review covers the total number of secondary metabolites reported from genus *Heterorhabditis* till date with their biomedical potential (Stock et al., 2017; Sivaramakrishnan and Razia, 2021).

### Symbiotic Association of *Photorhabdus* With *Heterorhabditis*

**FIGURE 1:**
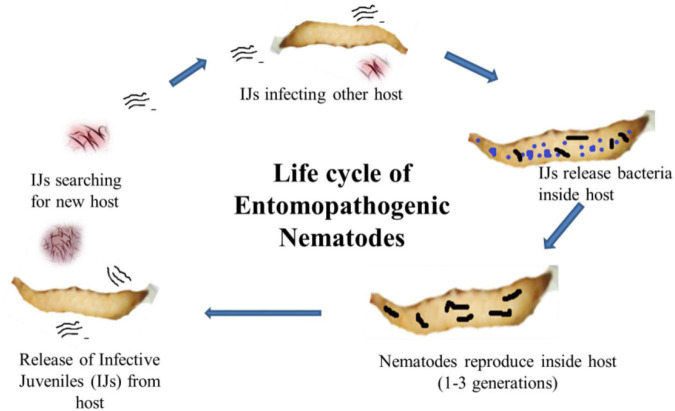
Life cycle of entomopathogenic nematodes showing infective juveniles (IJs) enter into the host body and release symbiotic bacteria from the gut to kill the host. The nematode reproduces inside the dead cadaver, and IJs are released into the soil in search of a new host.

*Photorhabdus* belongs to the family Enterobacteriaceae and is a Gram-negative bacterium symbiotically associated with the gut of *Heterorhabditis*, an entomopathogenic nematode (Forst et al., 1997). It is the only well-known terrestrial bioluminescent bacterium reported from the gut of IJs (infective juveniles) in EPNs. In the case of *Heterorhabditidae*, IJs do ambush, and they do not move in search of host and enter its body through natural openings (Figure 1). Once, it reaches the blood system of the host, the IJs regurgitates 50–200 bacterial cells (Ciche and Ensign, 2003) to conquer the immune system of the host and kill the host with septicemia. The IJs reproduce and multiply a few generations inside the host. The dead host raises the challenge of the *Photorhabdus* as it has to successfully compete with saprophytic scavengers, such as protists, other bacteria, nematodes, fungus, and even insects (Waterfield et al., 2009). In response, this symbiont produces a wide range of bioactives to cope up with the challenges for successful establishment and survival (Lulamba et al., 2021). Based on phenotypic characterization and DNA relatedness, *Photorhabdus* has been classified broadly into three species, i.e., *Photorhabdus luminescens*, *Photorhabdus asymbiotica*, and *Photorhabdus temperata.* This classification has been confirmed through limited microarray analysis (Marokhazi et al., 2003), genomic studies, and multilocus sequence typing (Duchaud et al., 2003; Gerrard et al., 2004). More recently, Machado et al. (2018) have proposed *Photorhabdus* subspecies to the species level and described one novel *Photorhabdus bodei* sp. and new *Photorhabdus laumonii* subspecies based on whole genome study. *Photorhabdus* species are facultative anaerobic and highly motile rods (Peel et al., 1999). All species produce a unique thin line of annular hemolysis on blood agar and grow well at 28°C. The clinical isolates grow from a temperature range of 37–42°C (Hapeshi et al., 2020). All the strains show bioluminescence on both liquid medium and agar plates, which peaks at exponential phase. The development of *Heterorhabditis* requires exogenous sterols (Chitwood, 1999), which are provided by the *Photorhabdus* to ensure successful symbiosis. *Photorhabdus* produces iso-branched fatty acids (BCFAs) through bkdABC operon (Joyce et al., 2008) for the nematode partner. For nematode nutrition, *Photorhabdus* produces CipA, CipB (crystalline inclusion proteins), and secondary metabolites, such as stilbene (ST), called 3,5-dihydroxy-4-isopropylstilbene (Joyce et al., 2008). *Photorhabdus* is the only non-plant organism that produces STs, a polyketide molecule. The biochemical pathway for the production of ST is different from that of plants and is well characterized (Williams et al., 2005; Joyce et al., 2008). InPtNC19, phosphopantethienyl (PPANT) transferase (ngrA gene) is vital for secondary metabolite synthesis, as well as nematode growth and reproduction (Ciche et al., 2001). In *Photorhabdus*, almost 6% genome is occupied by genes involved in secondary metabolite production, which is much greater than that of *Streptomyces*, the model organism for the production of secondary metabolite (Duchaud et al., 2003). *Streptomyces* uses 3.8% genome only and is a source of more than 90% clinically important antibiotics (Waterfield et al., 2009). This emphasizes the significant potential of *Photorhabdus* as a source of novel bioactive compounds. Studies have reported that *Photorhabdus* expresses two virulence factors, i.e., Tca (toxin complex A) and PrtA (metalloprotease) (Daborn et al., 2001; Silva et al., 2002). Recently, studies have also reported antimicrobial activities of compounds isolated from *Photorhabdus* species strain ETL in association with *Heterorhabditis zealandica* (Lulamba et al., 2021).

**FIGURE 2:**
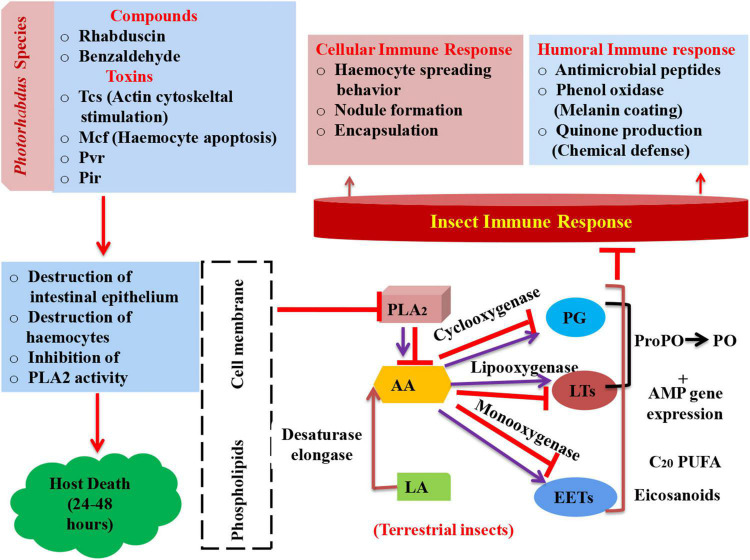
Schematic representation of insect’s immune response and mode of action of Photorhabdus compounds in arresting immune system leading to mortality (PLA2, phospholipase A2; AA, arachidonic acid; LA, linoleic acid; PG, prostaglandin; LTs, leukotrienes; EETs, epoxyeicosatrienoic acids; ProPO, prophenoloxidase; AMP, antimicrobial peptides; PO, phenol oxidase; Tcs, toxin complexes; Mcf, make caterpillars floppy; Pvc, Photorhabdus virulence cassettes; Pir, insect-related protein).

An overview of general mode of action of these compounds against different insect pest is shown in Figure 2.

## *Photorhabdus* as Source of Antiprotozoal Compounds

Protozoal parasitism is widespread among cats, dogs, and even humans (Baneth and Solano, 2020). Protozoans have caused a global health problem with diseases, such as malaria, giardiasis, trichomoniasis, trypanosomiasis, and leishmaniasis. Major disease-causing protozoans are *Leishmania mexicana*, *Trypanosoma cruzi*, *Plasmodium falciparum*, *Trichomonas vaginalis*, and *Giardia intestinalis* (Lee et al., 2019). These parasites spread worldwide infection due to poor sanitary and unhygienic conditions in developing countries (Garcia et al., 2003; Renslo and McKerrow, 2006; Pozio, 2007). About 200 million people get infected with malaria due to *Plasmodium*, and about half a million die annually (Ashley et al., 2018). In 2018, WHO reported 99.7% malarial cases in African region, 50% in the South-East Asia Region, 71% in the Eastern Mediterranean, and 65% in the Western Pacific due to *Plasmodium falciparum.* The WHO reports that in 2019, 12% cases of *Trypanosoma brucei rhodesiense* were endemic to 13 countries of southern and eastern Africa. Globally, 6–7 million people are infected with *Trypanosoma cruzi*, a protozoal parasite that causes deadly disease, such as American trypanosomiasis or Chagas disease (World Health Organization [WHO], 2018, 2019). This shows that antiprotozoal drugs lose efficiency to control the disease due to drug resistance and its toxicity. This has necessitated the need to find new source of antiprotozoal drugs. In this race, *Photorhabdus*, a nematode symbiont, has emerged as a reliable source of antiprotozoal compounds. Some of the antiprotozoal bioactive compounds isolated from *Photorhabdus* are given below.

### Phototemtide A (1)

A new cyclic lipopeptide, phototemtide A (1), was isolated from *Escherichia coli* expressing the biosynthetic gene cluster pttABC from *Photorhabdus temperata* Meg1. This new cyclic lipopeptide has three more minor derivatives. It has been reported that this peptide has weak antiprotozoal activity with IC_50_ = 9.8 μM, against deadly plasmodium species *Plasmodium falciparum* (Zhao et al., 2020). New peptide drugs have gained attention due to their easy synthesis, low toxicity, fewer side effects, and rapid elimination (Du et al., 2015). The quantity of market active constituents is very less due to unpleasant side effects and observed resistance (Weinke et al., 1991; Steketee et al., 1996; Plowe, 2005; Uhlemann and Krishna, 2005). This compound is selectively effective against *Plasmodium falciparum* and maybe a potential antiprotozoal alternative in the future.

### Photoditritide (2)

A cyclic peptide photoditritide, which was isolated from *Photorhabdus* temperata Meg1, which contains two rare amino acid D-homoarginine residues and encoded by pdtS gene. The gene pdtS codes for non-ribosomal peptide synthetase having six modules with 18 domains in all. It showed weak antiprotozoal activity against causative agent of African sleeping sickness, i.e., *Trypanosoma brucei rhodesiense* with IC_50_ = 13 μM (Zhao et al., 2019). Peptides containing homoarginine, a non-proteinogenic amino acid, have been reported from many marine organisms, such as cyanobacteria, actinomycetes, and sponge, but photoditritide is the only example of peptide containing homoarginine derived from entomopathogenic bacteria (Bonnington et al., 1997; Saito et al., 2001; Cha et al., 2012; Zhao et al., 2019).

### Isopropylstilbene

This is a class of natural products produced by *Photorhabdus luminescens* TT01. Many derivatives of this class have been synthesized by modifying PAL gene and antB gene in *Photorhabdus luminescens* mutant (BMM901), such as cyclohexanedione (CHD) and dialkylresorcinol (DAR) derivatives (Joyce et al., 2008; Kronenwerth et al., 2014). The biosynthesis of isopropylstilbene from *Photorhabdus* varies from plant stilbene biosynthesis, whereby two acyl moieties become condensed to form a resorcinol ring (Ferrer et al., 2008; Joyce et al., 2008; Fuchs et al., 2013). Synthetic derivatives, such as 12–14 and 1 and 6, were reported to be more effective against *Trypanosoma cruzi* (causes Chagas disease) and *Plasmodium falciparum* (causes malaria), respectively. Some chemically synthesized derivatives were more active against *Trypanosoma cruzi* (LC_50_ = 8.80 μM) and *Leishmania donovani* (LC_50_ = 3.71 μM) (Kronenwerth et al., 2014). This potent class of antiprotozoal compounds can meet the needs of pharmaceutical sector in the future.

### Kolossin A (3)

**FIGURE 3:**
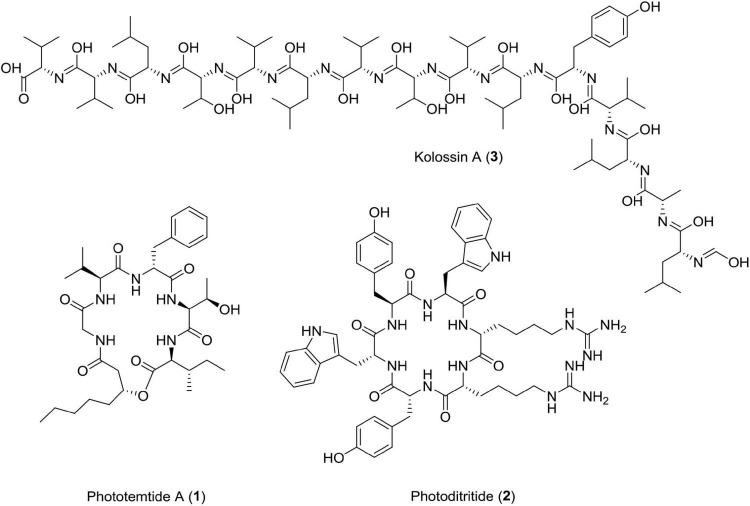
Chemical structure of antiprotozoal compounds **(1–3)**.

*Photorhabdus luminescens*, an entomopathogenic bacterium bears a flag of producing the largest and continuous non-ribosomal peptide synthetase among bacteria (Bode et al., 2015). It possesses a fully functional uninterrupted gene that could produce 15 consecutive modules encoded by kol gene (plu2670, 49.1 kbp). Many gene clusters of its genome are involved in biosynthesis of diverse natural products (Duchaud et al., 2003; Yin et al., 2015). *Photorhabdus luminescens* produces kolossin A, a d-/l-pentadecapeptide biosynthetic product of non-ribosomal peptide synthetase. It has been reported that stereoisomer of Kolossin A, displays high activity against the causative agent of African sleeping sickness, i.e., *Trypanosoma brucei rhodesiense (IC_50_* = *2.7*μ*M*), *Plasmodium falciparum (IC_50_* = *16.1*μ*M*), and *T. brucei r. (IC_50_* = *8.9*μ*M*) (Bode et al., 2015). This class of antiprotozoal compounds can prove to be useful for drug discovery against deadly protozoal diseases in the near future (Figure 3 and Table 1).

**TABLE 1:** List of bioactive compounds isolated from different species of *Heterorhabditis* and *Photorhabdus*.

Bacterial/nematode symbiont	Compound	Class of compound	Target organism/cell lines	Effective inhibitory concentration	References
**Antiprotozoal activity**					
*Photorhabdus temperata* Meg1	Phototemtide (A)(1)	Cyclic lipopeptide	*Plasmodium falciparum*	IC50 = 9.8 μM	Zhao et al., 2020
			*Trypanosoma brucei rhodesiense*	IC50 = 62 μM	
			*Trypanosoma cruzi*	IC50 = 83 μM	
			*Leishmania donovani*	IC50 > 100 μM	
*Photorhabdus temperata* Meg1	Photoditritide	Cyclic peptide	*Trypanosoma brucei rhodesiense*	IC50 = 13 μM	Zhao et al., 2019
			*Plasmodium falciparum*	IC50 = 27 μM	
			*Trypanosoma cruzi*	IC50 = 71 μM	
			*Leishmania donovani*	IC50 > 100 μM	
*Photorhabdus luminescens* TT01/*Heterorhabditis* sp.	Isopropylstilbene		*Trypanosoma cruzi*	LC50 = 8.80 μM	Kronenwerth et al., 2014
			*Leishmania donovani*	LC50 = 3.71 μM	
*Photorhabdus luminescens* TT01/*Heterorhabditis* sp.	Kolossin A and its stereoisomers	Pentadecapeptide	*Trypanosoma brucei rhodesiense*	IC50 = 2.7 μM	Bode et al., 2015
			*Plasmodium falciparum*	IC50 = 16.1 μM	
**Antifungal activity**					
*Photorhabdus temperata* M1021/*Heterorhabditis* sp.	Benzaldehyde	Aromatic aldehyde	*Phytophthora capsici*	IC50 = 5.7 mM	Ullah et al., 2015
			*Corynespora cassiicola*	IC50 = 8.1 mM	
			*Rhizoctonia solani*	IC50 = 6.0 mM	
*Photorhabdus temperata* SN259/*Heterorhabditis* sp.	Stilbene derivatives	Phenolics	*Pythium aphanidermatum*	EC50 = 2.8 and 2.7 μg/ml	Shi et al., 2017
*Photorhabdus luminescens*/*Heterorhabditis bacteriophora*	Trans-cinnamic acid	Unsaturated carboxylic acid	*Colletrotrichum gloesporioides*	10 and 100 μg/ml-1	Bock et al., 2014
			*Colletotrichum fragariae*		
**Cytotoxic compounds**					
*Photorhabdus asymbiotica*/*Heterorhabditis* sp.	Luminmycin D		Human pancreatic cell lines	IC50 = 0.11 μM	Theodore et al., 2012
*Photorhabdus luminescens*/*Heterorhabditis megidis*	Epoxide 1	Cyclic ether	T-cell leukemia	GI50 = 0.42 μM	Hu et al., 2006
			Lung cancer (H460)	GI50 = 0.63 μM	
			Breast cancer (MCF-7 wt)	GI50 = 2.14 μM	
*Photorhabdus luminescens* TT01/*Heterorhabditis* sp.	Lumizinones A		Calpain inhibitor	IC50 = 3.9 μM	Park and Crawford, 2016
**Anti-neuroinflammatory and neuroprotective compounds**					
*Photorhabdus temperata*/*Heterorhabditis* sp.	Anthraquinones 1,3,8-trihydroxy-9,10-anthraquinon (A)	Polycyclic aromatic hydrocarbon	Hippocampal neuronal cells (HT22)	75 μM	Yang et al., 2018
	3,8-Dihydroxy-1-methoxy-9,10-anthraquinon (B).		Microglial cells (BV2)	10 ng/ml	
**Proteasome inhibitors**					
*Photorhabdus luminescens*/*Heterorhabditis* sp.	Cepafungin I (CepI)		Proteosomal degradation	IC50 = 4.0 nM	Stein et al., 2012
*Photorhabdus asymbiotica*/*Heterorhabditis* sp.	Luminmycin D			IC50 = 0.38 μM	Theodore et al., 2012
**Antibacterial activity**					
*Photorhabdus temperata* M1021/*Heterorhabditis* sp.	Benzaldehyde	Aromatic aldehyde	*Bacillus anthracis* RSC-9	IC50 = 5.0 mM	Ullah et al., 2015
			*Pantoea conspicua* RSC-6	IC50 = 6.1 mM	
			*Enterobacter cowanii* RSC-3	IC50 = 4.5 mM	
			*Citrobacter youngae* RSC-5	IC50 = 7 mM	
			*Bacillus aryabhattai* RSC-7	IC50 = 4.0 mM	
*Photorhabdus temperata* Meg1/*Heterorhabditis* sp.	Photoditritide	Cyclic peptide	*Micrococcus luteus*	MIC = 3.0 μM	Zhao et al., 2019
*Photorhabdus luminescens*/*Heterorhabditis megidis*	Epoxide1	Cyclic ether	*Bacillus subtilis*	MICs = 12.5 μg/ml	Hu et al., 2006
			*Escherichia coli*	MICs = 6.25 μg/ml	
			*Staphylococcus aureus* (RN4220)	MICs = 6.25 μg/ml	
			*Staphylococcus epidermis* and *Streptococcus pyogenes* (ATCC 19615)	MICs = 12.5 μg/ml	
**Insecticidal compounds/phenoloxidase inhibitor (PO)**					
*Photorhabdus temperata* M1021/*Heterorhabditis* sp.	Benzaldehyde	Aromatic aldehyde	*Galleria mellonella*	Inhibit PO at 8 mM	Ullah et al., 2015
*Photorhabdus luminescens*/*Heterorhabditis* sp.	Rhabduscin		*Galleria mellonella*	Inhibit PO at 15 mM	Crawford et al., 2012
*Photorhabdus temperata* M1021/*Heterorhabditis* sp.	Ethyl acetate (EtOAc)	Esters	*Galleria mellonella*	Inhibit PO (60% activity)	Ullah et al., 2014a
	Phthalic acid (1,2-benzenedicarboxylic acid) (4)	Aromatic dicarboxylic acid		Inhibit PO (74% activity)	
*Photorhabdus luminescens*/*Heterorhabditis* sp.	(E)-1,3-dihydroxy-2-(isopropyl)-5-(2-phenylethenyl) benzene (ST)		*Manduca sexta*	275 μg/ml	Eleftherianos et al., 2007
**Plant growth regulators**					
*Photorhabdus temperate* M1021/*Heterorhabditis* sp.	Gibberellins GA1, GA3, GA4, and GA7	Diterpenes	*Oryza sativa*		Ullah et al., 2014b

## *Photorhabdus* as Source of Antifungal Compounds

Many fungal strains cause huge losses to crops, such as cereals and vegetables, and pose a serious threat to food security across the globe (Savary et al., 2006). Postharvest losses to vegetables and fruits due to many fungal pathogens lead to rotten crops and mycotoxin production to harm animals as well as humans (Saremi and Okhovvat, 2006; Bai et al., 2013). Huge loss to the crop has been caused by *Fusarium oxysporum* and *Pythium aphanidermatum due to postharvest decay* (Saremi and Okhovvat, 2006). Studies have reported antifungal activity of different compounds produced by entomopathogenic nematodes (Cimen et al., 2021). Some antifungal compounds produced by *Photorhabdus* species are listed below.

### Benzaldehyde (4)

*Photorhabdus Temperata* M1021 Produces Benzaldehyde as an Insecticidal, Antimicrobial, and Antioxidant Compound.

Antimicrobial activity was assessed by MIC values ranging from 6 to 10 mM for bacterial strains and 6–10 mM for fungal strains, i.e., *Phytophthora capsici* (IC_50_ = 5.7 mM), *Corynespora cassiicola* (IC_50_ = 8.1 mM), and *Rhizoctonia solani* (IC_50_ = 6.0 mM) (Ullah et al., 2015). Studies claim that the interaction of benzaldehyde with the cell surface triggers cell membrane disintegration and intracellular constituent release, which leads to cell death (Cheng et al., 2009).

### Stilbene Derivatives (5–9)

These classes of compounds are phenolics. It has been reported that seven derivatives of stilbene, i.e., 3-hydroxy-2-isopropyl-5-phenethylphenyl carbamate,2-(1-hydroxypropan-2-yl)-5-[(E)-2-phenylethenyl]benzene-1,3-diol, 2-(1-hydroxypropan-2-yl)-5-[2-phenylethyl]benzene-1,3-diol, 2-ethyl-5-(2-phenylethyl) benzene-1,3-diol, 2-isopropyl-5-[2-phenylethyl]benzene-1, 3-diol, 2-isopropyl-5- [(E)-2-phenylethenyl]benzene-1,3-diol, and 2-ethyl-5-[(E)-2-phenylethenyl]benzene-1,3-diol showed antifungal activity against four phytopathogenic fungi, such as *Rhizoctonia solani* Kuhn, *Pythium aphanidermatum*, *Fusarium oxysporum*, and *Exserohilum turcicum* (Paul et al., 1981; Fuchs et al., 2013). Out of these seven derivatives, 3-hydroxy-2-isopropyl-5-phenethylphenyl carbamate strongly inhibits *Pythium aphanidermatum* mycelium at EC_50_ = 2.8 and 2.7 μg/ml, respectively. The presence of acyl amino group in the former and isopropyl group in the latter contributed to inhibitory activity of these compounds.

### Trans-Cinnamic Acid

**FIGURE 4:**
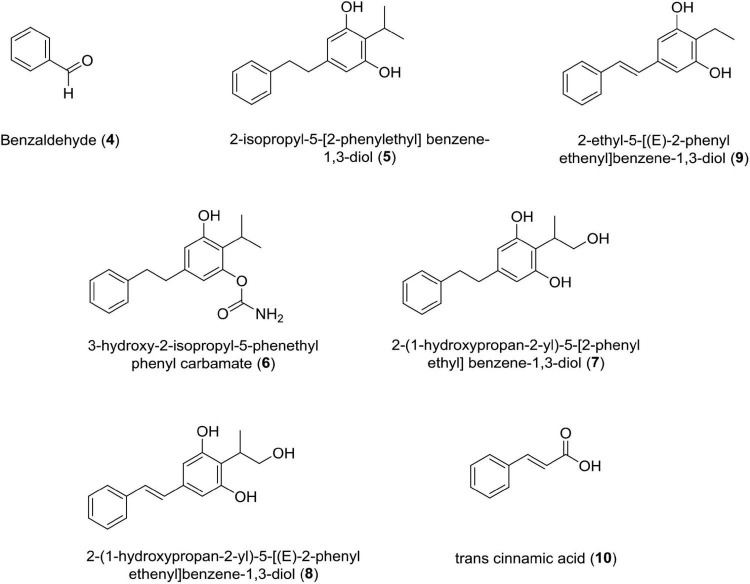
Chemical structure of antifungal compounds **(4–10)**.

This class of compound is a small molecule possessing antibiotic properties and food preservative (US patent doc numbers 6036986; 6042861) (Si et al., 2006; Wong et al., 2008; Chen et al., 2011; Hakkim et al., 2012). It has been reported that TCA is a necessary precursor for biosynthesis of the antibiotic stilbene (Williams et al., 2005; Eleftherianos et al., 2007; Chalabaev et al., 2008). Studies have established that TCA produced by *Photorhabdus luminescens* shows antimycotic activity against two important plant pathogens belonging to two fungal genera *Colletotrichum* and *Fusicladium*. *In vitro* studies have confirmed the toxicity of TCA against *Colletotrichum gloeosporioides*, *Colletotrichum acutatum*, *Colletotrichum fragariae*, and *Fusicladium effusum* (Bock et al., 2014). Recently, researchers have discovered phenylalanine ammonia-lyase (PAL) gene from *Photorhabdus luminescens* DSM 3368, which has enhanced the value of this strain for the production of TCA at commercial level (Zhang et al., 2021; Figure 4 and Table 1).

## *Photorhabdus* as Source of Cytotoxic Compounds

Cancer, the uncontrolled division of cells, claims more than 8 million lives annually and is the second leading cause of deaths globally (Tarver, 2012; Bray et al., 2013). Major cancer types reported amounts to 1.61 million (lung cancer), 1.38 million (breast cancer), and 1.23 million (colorectal cancers) (Ferlay et al., 2010). Among men, prostate, lung, colorectal, liver, and stomach cancer, whereas among women breast, lung, colorectal, thyroid, and cervical cancer are very common (World Health Organization [WHO], 2021). Pancreatic cancer is reported as highly lethal (Kamisawa et al., 2016). The hallmark features of cancer includes cancer cell ability to induce angiogenesis, evade apoptosis, replicate limitlessly, insensitivity to antigrowth signals, self-sufficient production of growth signals, tissue invasion, and metastasis (Hanahan and Weinberg, 2011; Merdad et al., 2014; Pérez, 2014; Snellenberg et al., 2014; Courtnay et al., 2015; Jiang et al., 2015; Mar et al., 2015; Frink et al., 2016).

### Glidobactins (11–15)

This novel compound belongs to the luminmycin metabolite family and is produced by *Photorhabdus asymbiotica* in laboratory culture. *Photorhabdus asymbiotica* has a unique, biphasic lifestyle as both a symbiotic and pathogenic bacterium. Genomic analysis revealed several synthetic gene clusters capable of producing secondary metabolites. *Photorhabdus asymbiotica* can produce cytotoxic derivatives, such as glidobactin A, luminmycin D, and luminmycin A. These compounds show cytotoxicity against pancreatic cells (IC_50_ = 0.11 μM) and inhibit proteasome (IC_50_ = 0.38 μM) (Theodore et al., 2012). Sequencing and annotation of the *Photorhabdus asymbiotica* (ATCC43949) genome have also been reported (Wilkinson et al., 2009). Recently, studies have reported cepafungin 1 and (GLNPs) glidobactin A produced by *Photorhabuds laumondii* as potent anticancer agents (Zhao et al., 2021).

### Epoxide 1

This novel compound epoxide 1, is also known as 2-isopropyl-5-(3-phenyl-oxiranyl)-benzene-1,3-diol. It was isolated from *Galleria mellonella* larvae infected with *Photorhabdus luminescens* C9-*Heterorhabditis megidis* 90 symbiont complexes (tripartite interaction, insect-nematode, and bacterium). Epoxide 1 has been derived from 2-isopropyl-5-(2-phenylethenyl)-benzene-1,3-diol (Hu et al., 1997, 1998). Epoxide 1 was active against *Bacillus subtilis*, *Escherichia coli*, *Streptococcus pyogenes*, and a drug-resistant, clinical strain of *Staphylococcus aureus* (RN4220) with minimum inhibitory concentrations in the range of 6.25–12.5 μg/ml. Epoxide 1 was cytotoxic against human cancer cell lines, MCF-7 wt, H460, and Jurkat, with GI (50) of 2.14, 0.63, and 0.42 μM, respectively, but was less toxic on normal, mouse splenic lymphocytes with a GI (50) of 45.00 μM (Hu et al., 2006).

### Lumizinones A (16)

**FIGURE 5:**
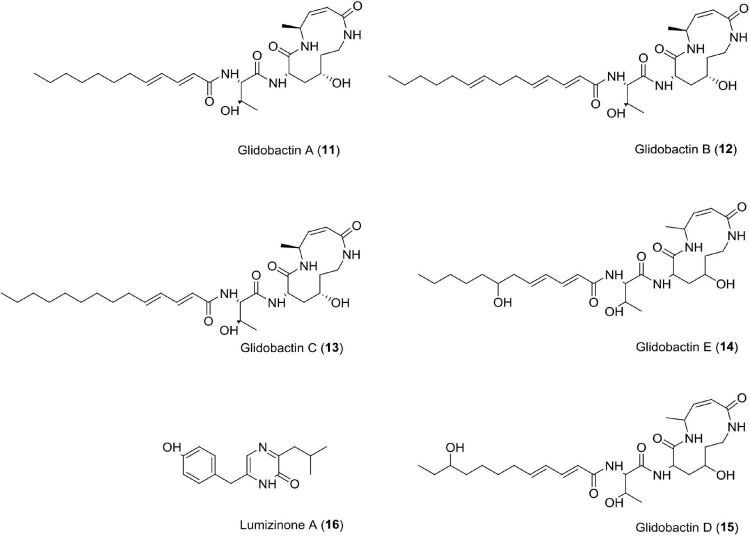
Chemical structure of cytotoxic compounds **(11–16)**.

Lumizinones are produced exclusively from the pathogenic form, a phenotypic variant of *Photorhabdus*, which is associated with insect pathogenesis and nematode development. *Photorhabdus*. This compound inhibits calpain protease activity at IC_50_ = 3.9 μM (Park and Crawford, 2016). Calpain is a heterodimer having two subunits, i.e., regulatory subunit (30 kDa) and catalytic subunit (80 kDa). It belongs to the intracellular cysteine proteases family, Ca^2+^-dependent, and distributed in the cytoplasm of cells and tissues in eukaryotes (Sato and Kawashima, 2001; Goll et al., 2003; Park and Crawford, 2016). Calpains are associated with cancer like schwannomas and meningiomas, renal cell carcinoma, colorectal adenocarcinoma, squamous carcinomas of the skin, prostate cancer, endometrial cancer, uterine sarcomas and carcinosarcomas, uterine cervical, neoplasiamelanoma, gastric cancer, laryngeal, colorectal, and pancreatic cancer (Kimura et al., 1998; Braun et al., 1999; Yoshikawa et al., 2000; Mamoune et al., 2003; Reichrath et al., 2003; Rios-Doria et al., 2003; Lakshmikuttyamma et al., 2004; Frances et al., 2007; Lee et al., 2007, 2008; Moretti et al., 2009; Fong et al., 2010; Salehin et al., 2010; Moreno et al., 2011; Figure 5 and Table 1).

## *Photorhabdus* as Source of Antineuroinflammatory and Neuroprotective Compounds

### Anthraquinones (17–18)

These quinones are derived from anthracene and are widely used in cosmetics, food, dyes, and pharmaceuticals. Over the years, many anthraquinone derivatives have been identified from bacteria, plants, insects, and fungi having a wide array of bioactivity like anticancer, laxation, neuroprotective, antimalaria, and anti-inflammatory effects (Bonadonna et al., 1969; Monks et al., 1992; Van Gorkom et al., 2002; Pankewitz et al., 2007; Wang et al., 2007; Batista et al., 2009; Kim et al., 2009; Gessler et al., 2013; Lim et al., 2017; Wu et al., 2017).

**FIGURE 6:**
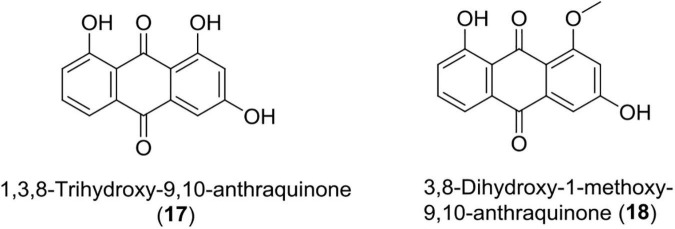
Chemical structure of anti-inflammatory compounds **(17–18)**.

Recently, *Photorhabdus temperata* has been reported as a source of anti-neuroinflammatory and neuroprotective drug leads, such as 1, 3, 8-trihydroxy-9, 10-anthraquinone, and 3, 8-dihydroxy-1-methoxy-9, 10-anthraquinone. An earlier compound has shown significant protection of hippocampal neuronal cells (HT22) at 75 μM in mouse against glutamate-induced cell death (5 mM) caused *via* lipid peroxidation, Ca^2+^ influx, and inhibiting reactive oxygen species production. Both the compounds have also been reported to suppress neuroinflammation induced by interferon γ in microglial cells (BV2) of mouse at a concentration of 10 ng/ml through reduction of interleukin-6, TNF (tumor necrosis factor-α), and nitric oxide (Yang et al., 2018; Figure 6 and Table 1).

## *Photorhabdus* as Source of Proteasome Inhibitor Compounds

Proteasome is a proteolytic complex having hollow cylinder and single torus protein described by the Harris group in 1970 (Harris, 1971). It is responsible for ubiquitinated protein degradation and has been a ray of hope to stop proliferation of malignant cells and cancer/multiple myeloma (Voorhees et al., 2003; Shah and Orlowski, 2009; Crawford et al., 2011). The function of proteosome is associated with ATP-dependent degradation of intracellular proteins, specifically having a polyubiquitin chain (Ciehanover et al., 1978; Ciechanover, 1998). Proteosome inhibitors are short peptides having a covalently bonded group of atoms called pharmacophore, which binds to proteosome at its catalytic sites and inhibits proteasome function (Kisselev et al., 2012). It is useful in treating multiple myeloma (MM), characterized by accumulation of pathological clonal plasma cells in bone marrow (BM) and a huge amount of monoclonal immunoglobulin (Ig) (Kyle and Rajkumar, 2009). *Photorhabdus luminescens* has emerged as a new source of this remarkable compound as explained below.

### Cepafungin I

It is a 12-member macrolactam ring system that is linked with a fatty acid tail, terminally branched, and having additional methyl moiety. This compound has been reported to be produced by *Photorhabdus luminescens*. This compound is similar to one of the strongest proteasome inhibitors Glidobactin A (GlbA) (Oka et al., 1988; Shoji et al., 1990; Groll et al., 2008).

### Luminmycin D

*Photorhabdus asymbiotica*-produced compound luminmycin D is also a potent proteasome inhibitor at IC_50_ value equal to 0.38 μM (Theodore et al., 2012).

## *Photorhabdus* as Source of Antibacterial Compounds

### Benzaldehyde (4)

This is a simplest aromatic aldehyde made up of benzene ring and formyl components. *Photorhabdus temperata* M1021 has been reported to produce this compound (Ullah et al., 2015). This compound shows antimicrobial properties against *Bacillus anthracis* RSC-9 (IC_50_ = 5.0 mM), *Pantoea conspicua* RSC-6 (IC_50_ = 6.1 *m*M), *Enterobacter cowanii* RSC-3 (IC_50_ = 4.5 mM), *Citrobacter youngae* RSC-5 (IC_50_ = 7 mM), and *Bacillus aryabhattai* RSC-7 (IC_50_ = mM).

### Photoditritide (2)

This compound has been reported from *Photorhabdus temperata Meg1*. This compound shows antimicrobial potential against bacterium *Micrococcus luteus* at an MIC value equal to 3.0 μM (Zhao et al., 2019).

### Epoxide1

This compound has been isolated from *Photorhabdus luminescens*. Studies have reported that this compound (2-isopropyl-5-(3-phenyl-oxiranyl)-benzene-1, 3-diol) is active against *Escherichia coli*, *Streptococcus pyogenes*, *Bacillus subtilis*, and *Staphylococcus aureus* (RN4220) at an inhibitory concentration ranging from 6.25 to 12.5 μg/ml (Hu et al., 2006; Figures 3, 4 and Table 1).

## *Photorhabdus* as a Source of Insecticidal Compounds and Phenoloxidase Inhibition

### Benzaldehyde (4)

This is an insecticidal compound produced by *Photorhabdus temperata* M1021. Studies have shown that it caused 100% mortality in *G. mellonella* at 8 mM concentration. It is toxic to insects and inhibited PO activity from 15 to 80% at different concentrations (Ullah et al., 2015).

### Rhabduscin (19)

This class of molecule is an amidoglycosyl- and vinyl-isonitrile-functionalized tyrosine derivative produced by *Photorhabdus luminescens*. This compound targets the innate immune system of the insect through a key component called phenoloxidase in *G. mellonella* and has emerged as a potential lead for the formation of insecticides (Crawford et al., 2012).

### Phurealipids

This compound is a simple urea compound produced by *Photorhabdus luminescens*. Studies have shown that it inhibits juvenile hormone epoxide hydrolase (JHEH), which is an important enzyme in the growth and development of insects. This makes it a more suitable chemical with insecticidal properties (Nollmann et al., 2015b).

### (E)-1,3-Dihydroxy-2-(Isopropyl)-5(2-Phenylethenyl) Benzene (ST)

This is a small molecule having antibiotic properties and is produced by *Photorhabdus luminescens* in both *in vivo* as well as *in vitro* conditions. Cinnamic acid is a precursor of ST and is catalyzed by an enzyme encoded by stlA gene, which is a *Photorhabdus* gene (Eleftherianos et al., 2007). Studies have shown that this compound shows a wide range of antimicrobial activity and defends dead insects from microbe invasion (Hu and Webster, 2000; Williams et al., 2005).

### 3,5-Dihydroxy4-Isopropylstilbene

This class of compound has been isolated from *Photorhabdus luminescens* in dead *G. mellonella* larvae. This compound minimizes the competition between different microbes by inhibiting the growth of a wide range of bacterium (Li et al., 1995; Hu et al., 1998). This hypothesis is well supported in *in vitro* studies, but *in vivo* shreds of evidence are very less and have been questioned by Dutky (1959, 1974), Paul et al. (1981), Akhurst (1982), Li et al. (1995), Forst and Nealson (1996) and Jarosz (1996).

### Phthalic Acid (20)

**FIGURE 7:**
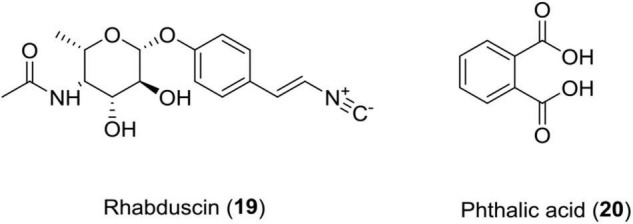
Chemical structure of insecticidal compounds **(19–20)**.

This compound was isolated from *Photorhabdus temperata* and has shown reliable insecticidal activity against *G. mellonella* (Figure 7 and Table 1).

### Mode of Action of Insecticidal Compounds

Phospholipase A_2_ (PLA_2_) plays a key role in imparting immunity to insects as it releases arachidonic acid (AA) *via* its catalytic activity from phospholipids (Burke and Dennis, 2009). AA acts as precursor molecule in synthesis of eicosanoid. Eicosanoids are oxygenated C_20_ polyunsaturated fatty acids. These eicosanoids are grouped into three main categories, namely, prostaglandins (PGs), epoxyeicosatrienoic acids (EETs), and leukotrienes (LTs) (Stanley, 2014). Cyclooxygenase, monooxygenases, and lipoxygenase oxygenate AA into PGs, EETs, and LTs, respectively (Stanley and Kim, 2019). However, in the case of terrestrial insects, linoleic acid (LA) is present, which becomes converted into AA with the help of elongase and desaturase enzymes (Hasan et al., 2019). Furthermore, PGs and LTs, in turn, activate phenol oxidase (PO) *via* formation of proPo and antimicrobial gene expression (AMP) (Shrestha and Kim, 2008; Shrestha et al., 2011). This PO catalyzes the process of melanization, which is a crucial event that leads to insect mortality (Castillo et al., 2011). Bioactive compounds rhabduscin have the ability to destruct the host’s epithelium and inhibit the catalytic activity of PLA_2_ (Figure 2).

## *Photorhabdus* as Source of Plant Growth-Regulator Compounds

### Gibberellins

Gibberellins represent a group of tetracyclic compounds, diterpenoid compounds consisting of four isoprene units having an ent-gibberellane ring skeleton. It plays an essential role in germination, stem elongation, flowering, dormancy, sex expression, induction of enzymes, and senescence of fruit and leaf. This compound has been reported to be produced by *Photorhabdus temperata* M1021, a symbiont of entomopathogenic nematodes. Various bioactive GAs reported from *Photorhabdus temperata* M1021 through GC/MS-SIM analyses are GA1, GA3, GA4, GA7, GA9, GA12, and GA20 (Ullah et al., 2014b). GAs initiate cellular totipotency, seed germination, and plant growth (Großelindemann et al., 1992; Hedden and Kamiya, 1997; Table 1).

## Other Compounds

### Polyketide Pigments

These pigments are secondary metabolites having carbonyl and methylene groups, or have precursors with this group. Studies have reported these compounds from *Photorhabdus luminescens* strain TT01 and linked these compounds with antibiotic activity (Ffrench-Constant et al., 2003).

### Photorhabdicins—R-Type Pyocins (21)

**FIGURE 8:**
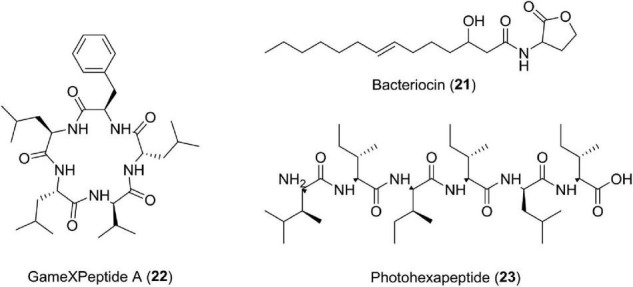
Chemical structure of other compounds **(21–23)**.

Bacteriocin is a protein, which is produced by one species of bacteria to inhibit some other strain. Studies have shown that *Photorhabdus aeruginosa* produces R-type pyocins having modified tail fibers, which strongly bind to bacterial surfaces and advocate antibacterial activity (Eleftherianos, 2009). Photorhabdicins are compounds having structures similar to R-type pyocins, have been reported from K122 and W14 strains of *Photorhabdus*, and possess a vital role against microbes (Ffrench-Constant et al., 2003) (Figure 8).

### Lumicins—S-Type Pyocins

This is another type of bacteriocin reported to be produced by the W14 strain of *Photorhabdus luminescens* (Sharma et al., 2002). It carries many killer proteins to counter the effect of many other bacteria strains (Sharma et al., 2002).

### Carbapenem Antibiotics

It is a class of antibiotics produced *via* biosynthetic pathways in cephamycins, penicillins, and cephalosporins (Williamson et al., 1985; Bycroft et al., 1988). It was reported that *Photorhabdus luminescens* TT01 possess a group of eight genes (cpmH to cpmA), which are responsible for carbapenem-like antibiotic production. This compound was found effective against *Escherichia coli*, *Enterobacter cloacae*, and *Klebsiella pneumonia* (Derzelle et al., 2002).

### Anthraquinone Metabolites

Many Anthraquinone (AQ) pigments have been reported from *Photorhabdus* (Richardson et al., 1988; Li et al., 1995). Two gene clusters (plu4186-plu4194) have been identified for the production of type II PKS, which is predicted to form an AQ heptaketide backbone (Duchaud et al., 2003). These compounds possess antibiotic potential (Richardson et al., 1988; Sztaricskai et al., 1992; Li et al., 1995; Brachmann et al., 2007). Two more anthraquinone derivatives, i.e., 1,3-dimethoxy-8-hydroxy-9,10-anthraquinone (major) and 3,8-dimethoxy-1-hydroxy9,10-anthraquinone (minor) have been reported from *Photorhabdus luminescens* (Li et al., 1995).

### Hydroxystilbene Compounds

Among the hydroxystilbene family, 1,3-dihydroxy-2-(isopropyl)-5-(2-phenylethenyl) benzene (ST) is an important molecule. This is a small multifunctional compound produced by only one bacterium, i.e., *Photorhabdus luminescens*, and shows antibacterial properties (Akhurst, 1982; Richardson et al., 1988; Li et al., 1995; Williams et al., 2005). ST epoxide, another related compound having powerful antibiotic potential, has also been reported from *Photorhabdus* species (Hu et al., 2006). stlA gene is responsible for the production of stilbene antibiotic molecules (Williams et al., 2005). Reports of antibiotic, 3,5-dihydroxy-4-isopropylstilbene from *Photorhabdus luminescens* are also available (Li et al., 1995).

### Cinnamic Acid

This compound is a precursor of the antibiotic 3,5-dihydroxy-4-isopropylstilbene (ST) (Williams et al., 2005; Eleftherianos et al., 2007). Antioxidant as well as antibacterial activities of this compound have been reported (Korkina, 2007). *Photorhabdus luminescens* studies have linked Hca enzyme with ST synthesis and CA utilization (Chalabaev et al., 2008).

### Photobactin

This compound is a catechol-type siderophore produced by *Photorhabdus luminescens* having the structure 2-(2,3-dihydroxyphenyl)-5-methyl-4,5-dihydro-oxazole-4-carboxylic acid [4-(2,3-dihydroxybenzoylamino)-butyl]-amide (Ciche et al., 2003). Purified photobactin also shows antibiotic activity (Paul et al., 1981; Akhurst, 1982; Richardson et al., 1988).

The bacteria of the genus *Photorhabdus* is also a source of many compounds having unknown functions or multifunctions. Studies have reported new pentapeptides known as GameXPeptides A (**22**) from *Photorhabdus luminescens* TTO1. These peptides are of unknown nature (Nollmann et al., 2015a). In *Photorhabdus asymbiotica* PB68.1, a library of photohexapeptides (**23**) has been generated after activating phpS, which is a silent gene. The photohexapeptide compound belongs to the rare linear D-/L-peptide family, which also includes feglymycin, kolossin (A), and gramicidin A (Zhao and Bode, 2019; Figure 8 and Table 1).

## Discussion and Conclusion

Natural products (NPs) possess enough structural complexity and scaffold diversity. Historically, NPs and their analogs have contributed a lot to the pharmacology sector. Inspite of this, NPs also possess challenges for drug discovery like technical barrier to isolation, characterization, screening, and optimization, which has a decline in their pursuit in the pharmaceutical industry since 1990s (Atanasov et al., 2021). Evolution has structurally optimized NPs to serve peculiar biological function, such as interaction (inter- and intraspecific competitions) and defense mechanisms, which emphasize their relevance in combating many diseases (Atanasov et al., 2015). Bioactive compound-rich NP pool covers a broad chemical space compared with a library of small synthetic molecules (Lachance et al., 2012). A wide array of these natural products produced by natural processes plays a vital role in symbiotic associations. Entomopathogenic nematodes possess a huge diversity and indicate a huge scope of research for natural products. The genus has attracted research interest in the past decade because it has emerged as a new group of biocontrol agents against pathogens of crop plants and as a new source of bioactive natural products. This article includes screening reports of bioactive compounds, which require further studies before clinical trials. Considering the current crisis in antibiotic resistance, the discovery of novel antibiotics is of great importance, and this association of *Heterorhabditis* and *Photorhabdus* might play a wider role in human survival in the twenty-first century.

However, there are still many aspects remaining to be studied. First, the genome sequencing of these bacteria revealed the presence of several predicted gene clusters. The exact roles of these are still unknown and need thorough investigation. So far, the main effort in this area has been devoted to isolation and structural determination. Second, the mining of these gene clusters with media manipulation and epigenetic approach for new natural products is still in its infant stage. Future research should set out to identify new selection markers, powerful promoters, and innovative approaches to tackle the low gene expression level and often extremely poor product yield. Finally, more efforts are needed in the studies of application as new biocontrol agents. A key factor for the success of this biological approach is the discovery of new microbial strains that can produce potent natural products with novel chemistry and modes of action.

## Author Contributions

All authors listed have made a substantial, direct, and intellectual contribution to the work, and approved it for publication.

## Conflict of Interest

The authors declare that the research was conducted in the absence of any commercial or financial relationships that could be construed as a potential conflict of interest.

## Publisher’s Note

All claims expressed in this article are solely those of the authors and do not necessarily represent those of their affiliated organizations, or those of the publisher, the editors and the reviewers. Any product that may be evaluated in this article, or claim that may be made by its manufacturer, is not guaranteed or endorsed by the publisher.
